# Guiding Midwives to Precept Students: Two Evidence‐Based Models

**DOI:** 10.1111/jmwh.70075

**Published:** 2026-01-14

**Authors:** Margaret C. Taylor, Hannah L. Diaz

**Affiliations:** ^1^ Vanderbilt University School of Nursing Nashville Tennessee

## Abstract

The success of midwifery education relies heavily on the time and expertise shared by experienced midwifery preceptors. Although there are several known and documented barriers to preceptor availability and willingness to participate in the education of future midwives, clinical education opportunities are crucial to midwifery education. Mentorship and training from preceptors not only benefit the students but also contributes to the development of a well‐prepared, compassionate midwifery workforce. Advocates for growing the US midwifery workforce point to the alarming rate of maternity health care deserts, in which families lack access to maternity health care providers and maternity care services. Midwives are the logical solution to fill this health care gap, but this requires the growth of midwifery education programs and clinical preceptors. Recognizing the importance of precepting and easing the role of the preceptor with recommended teaching methods can lighten the burden and make the precepting experience more beneficial. Health profession education has used various preceptor models to guide learning interactions, but there is not a specific model for midwifery clinical education. Midwives need standardized, reliable, and consistent methods that engage students, promote critical thinking skills, and create positive learning experiences. In this article, we propose and discuss the use of 2 evidence‐based models: the One‐Minute Preceptor method and the Summarize, Narrow, Analyze, Probe, Plan, Select method to facilitate and support the role of the preceptor. Investing in preceptors is an investment in the future of quality midwifery care.

## INTRODUCTION

When compared to other high‐income countries in the world, the United States has the highest rates of maternal mortality and morbidity, the lowest number of midwives, and declining access to maternity services.^1^ US national statistics for 2023 demonstrated that 97.7% of all births occurred in the hospital setting with physicians, with midwives being present for 12.6% of the total births both in and out of the hospital.^2^ The midwifery data identified and combined 2 types of midwives: certified nurse‐midwife or certified midwife and other midwife.^2^ These figures stand in direct contrast to birth care in similar industrialized countries such as the United Kingdom, where the majority of all births were attended by midwives.^1^ The Commonwealth Fund reports that the US rate of maternal deaths is more than double that of comparable high‐income countries.^1^ The most recent data from 2022 reported that there were 22 maternal deaths for every 100,000 live births in the United States, compared with 13.6 in New Zealand, and 5.5 in the United Kingdom.^1^ Additionally, many US communities are flagged as maternity care deserts, designated by a lack of access to maternity providers and services.^3^ It is estimated that 35% to 36% of counties in the United States meet this criteria, driving up rates of preterm birth and maternal mortality, and contributing to the country's poor standing globally. The development of a robust midwifery workforce is crucial to address maternity care access and the maternal morbidity and mortality crisis in the United States.^1^, ^3^, ^4^ However, midwifery growth depends on funding education programs to meet national standards and developing a strong network of clinical preceptors. Although legislative measures and program expansions exist, the shortage of clinical preceptors is a critical limiting factor.^4^, ^5^ This article examines the evidence‐based elements for expanding and improving clinical preceptorship skills using evidence‐based preceptor tools.

  

Continuing education (CE) is available for this article. To obtain CE online, please visit http://www.jmwhce.org. A CE form that includes the test questions is available in the print edition of this issue.

### Challenges in Precepting

Training well‐qualified midwives involves a combination of didactic and clinical education. In addition to mastery of evidence‐based content in the classroom, continued student success relies on the time, expertise, and generosity of midwifery preceptors. Motivation to precept may include personal and professional reasons, for example, the enjoyment of teaching, the contribution to the profession, and continuing education credit.^6^, ^7^ Despite the desire to provide clinical education to students, preceptors often face real‐world barriers such as time constraints, lack of institutional support, and a lack of clear preceptor role responsibilities.^6^, ^8^ Moreover, there is no consistent approach or model to prepare midwifery preceptors for daily student interactions. The American College of Nurse‐Midwives (ACNM) offers a preceptor training model, the Preceptor Education Program, free of charge through the ACNM website without requiring membership.^10^ This preceptor and student training was developed in Canada for use with professional students in the medical field. There is a total of 8 modules that include videos and interactive components that cover basic precepting, communication, and evaluation skills. However, working through the modules may take several hours, and busy clinicians may not have the time to complete the material.^6^ Although the material is comprehensive, the modules are generic to all health professionals and lack sufficient tailoring to the unique demands of midwifery that encompasses the midwifery model of person‐centered care.^10^ An additional resource is a members‐only preceptor training available through the National Organization of Nurse Practitioner Faculties.^11^

1 **QUICK POINTS**

✦ Preceptors are the key factor needed to grow the midwifery workforce in the United States.
✦ Easing the preceptor role with recommended methods can lighten the burden and increase willingness to precept for busy clinicians.
✦ The use of evidence‐based models, such as the One‐Minute Preceptor and Summarize, Narrow, Analyze, Probe, Plan, Select, provides standardized, reliable, and consistent formats for precepting midwifery students.

Midwives who assume preceptor roles need clear steps for managing the clinical learning process. Skills for interacting with students in a way that promotes learning are largely self‐taught, and preceptors rely on their own experiences as students to guide their teaching. Recent research by Blumenfeld et al^6^ found that midwives report being trained as clinicians rather than educators and that more support is needed to help them precept effectively. Likewise, Boyce et al^12^ reported one of the key barriers to successful precepting was a lack of education or training for preceptors prior to taking on a student, with clinicians desiring more specific guidance. Although there are numerous studies about clinical teaching models in medical and nursing literature,^13^ only 3 articles were found in the midwifery literature specific to the use of preceptor tools.^14^, ^15^, ^16^ These studies have advocated for the one‐minute preceptor (OMP) and the Summarize, Narrow, Analyze, Probe, Plan, Select (SNAPPS) resources.

For any method to be effective, the literature is clear that a supportive environment for students to engage in critical thinking is imperative. Creating a nurturing relationship is foundational to a successful student experience.^6^, ^17^ Positive clinical experiences also allow students to flourish and build confidence.^6^ Additionally, preceptors benefit from these affirmative relationships through improved confidence in their teaching abilities. Providing preceptors with better preparation and clinical teaching methods is one proposed avenue to improve preceptor–student interactions, thus setting the stage for more meaningful clinical experiences.^6^

## PROPOSED CLINICAL TEACHING METHODS

In the clinical space, students are expected to follow the midwifery management process as outlined in the ACNM Core Competencies.^18^ Initial steps include reviewing the chart, obtaining a history, performing a targeted examination, and developing an assessment. Beginning students present this information to the preceptor in an organized fashion and respond to clarifying questions as needed. Together, the preceptor and student then determine a plan of care. More advanced students would also present a list of differential diagnoses and develop an evidence‐based plan of care. This plan would include any diagnostic examination findings and physician consultation or collaboration as dictated by the clinical situation. Essential to this model is the student taking ownership of the process, using self‐reflection and a critical review of their care.

Although this framework provides a structure for student learning needs, the preceptor must also feel confident in their abilities to guide students in navigating the person‐centered aspects of midwifery care. Thus, the use of a structured precepting model or tool can provide real‐time guidance. Two of the most studied and used tools in medical and nursing literature are the OMP and SNAPPS.^12^, ^19^, ^20^ Lazarus^14^ has provided an overview on both methods, emphasizing that these clinical teaching strategies can enhance clinical efficacy while improving student experience. The goal of this article is to present these 2 evidence‐based models to better clarify and streamline the preceptor role, thereby improving the clinical experience for both the preceptor and the student. Both authors of the article have acted as preceptors for midwifery and other health professional students, and they both also function as educators and faculty within a midwifery program.

### The OMP

The OMP was developed by Neher et al for use in the family practice outpatient setting.^21^ It was designed to lead the student through the decision‐making process, with the entire approach taking 5 minutes or less.^21^ The OMP is teacher‐led and works well with students of any level, making it ideal for use with novice learners.^20^, ^21^ Composed of 5 micro‐skills, the goal of the OMP was to move clinical teaching away from lecturing and into discussions that fostered critical thinking. The 5 micro‐skills include (1) getting a commitment, (2) probing for supporting evidence, (3) teaching general rules, (4) reinforcing what was done right, and (5) correcting mistakes. The steps are typically followed in order with the student first choosing a diagnosis (commitment) and presenting supporting evidence. The preceptor then challenges the student to consider other possibilities (probes) before providing the general rules or practice pearls. Feedback is provided to the student on what was done well. Critiques or corrections are reserved for the final step, which is essential to providing positive student learning experiences.^17^, ^22^

Studies in both medicine and nursing have shown that the OMP has been well received in preceptor training programs resulting in improved preceptor–student feedback.^19^, ^23^, ^24^, ^25^ Gatewood et al^19^ conducted a multidisciplinary study of community clinicians who precepted nurse practitioner (NP) students to assess the OMP's effectiveness. The authors offered a two‐hour workshop to 36 community clinicians across disciplines. Using pre‐ and postsurveys, the authors found that the participants who used the OMP reported that the preceptor training influenced them to make changes in their teaching, promoting positive feedback and correction of mistakes for their students.

The effectiveness of a pilot training program for NPs using the OMP was conducted by Miura et al.^25^ The authors adapted and used several validated questionnaires to assess the effectiveness of the preceptor training program. The NPs who used the OMP training in their clinical teaching reported improvements in self‐confidence and in their experiences as educators with an improved willingness to serve as preceptors.^7^, ^25^ Although this was a small study of 9 NPs, these findings have important implications for education programs in recruiting and sustaining engaged preceptors.

In another article, Penwell^16^ used a case study approach to encourage certified professional midwives (CPMs) to use the OMP when precepting students. While the author supported the use of this method for CPM students, it is unclear whether a formal training program was created. To date, there is no published information on the outcomes of a preceptor training for midwives using OMP with the author's affiliated university.

In summary, the OMP is an organized format to guide the midwifery student through the patient assessment process in a structured manner.^21^ The expectations for novice versus advanced students need to be anticipated, as well as giving the student time to respond. The actions in Step 1 and Step 2 may overlap, especially for advanced students. Initially, the novice may be more inclined toward diagnosing commonly reported symptoms of pregnancy, whereas an advanced student can be expected to provide additional differential diagnoses. In probing the evidence, the preceptor can prompt the novice to think bigger before providing midwifery pearls. The OMP also provides steps for both positive and corrective feedback. Feedback tied to the specifics of a clinical situation is one of the most critical steps in the learning process.^22^ Although this method is teacher‐driven, the student can easily learn the process and be more prepared for the next case presentation. The OMP is one of the most widely adapted tools in health care settings, and while training is not required, preceptor training using this strategy has demonstrated benefits.^19^, ^25^, ^26^

### SNAPPS

The SNAPPS methodology was developed by Wolpaw and colleagues in 2003 and has been adapted for use in the medical setting for over 20 years.^27^ SNAPPS is comprised of 6 steps: (1) summarize briefly the history and findings; (2) narrow the differential to 2 or 3 relevant possibilities; (3) analyze the differential by comparing and contrasting the possibilities; (4) probe the preceptor by asking questions about uncertainties; (5) plan the management for the patient's medical issues; (6) select a case‐related issue for self‐directed learning.

The SNAPPS method was developed to move the student away from the traditional teacher‐controlled process in which the student reports the facts of the patient's case and answers the preceptor's questions.^27^ SNAPPS is a learner‐driven model that challenges students to present the case and take ownership of the diagnostic and management process. The student must provide multiple differential possibilities and then ask the preceptor probing questions to correct their diagnoses and move toward a management plan. This process gives the preceptor information about the student's level of knowledge and ability to assess the data. The student is not penalized for incorrect diagnosis or asking questions but rather encouraged to explore all possibilities.

In the original publication for this method, the authors conducted a pilot study of SNAPPS training faculty preceptors and students, then pairing them in the outpatient setting.^27^ Both the faculty and the students gave the method high marks because it changed the teacher‐student dynamics, resulting in more questions and interactive discussions compared to the traditional method. Several randomized control trials (RCTs) have since been conducted to evaluate the efficacy of SNAPPS for promoting critical thinking in the medical setting.^28^ Jain et al^29^ conducted an RCT in 2019 comparing the use of SNAPPS (intervention group) with traditional case presentation (control group) methods among 18 residents in a medical residency program. The SNAPPS group showed improved student diagnostic hypothesis and improved discussion of patient management while maintaining thoroughness. The study findings concluded that SNAPPS facilitated clinical discussions and clinical reasoning.

Another RCT by Shindala et al^30^ was conducted in 2024 to evaluate the effectiveness of SNAPPS among 30 Family Medicine residents and 6 faculty participants. The intervention group of 15 residents and all of the faculty received SNAPPS training. The participants in the SNAPPS group performed better in terms of time efficiency, number of case details covered, and reasoned diagnoses, as well as in asking questions and seeking information. The authors concluded that SNAPPS was a valuable tool to engage students’ critical thinking skills. Additionally, both the preceptors and the residents gave the method superior ratings.

The singular study examining the use of SNAPPS in midwifery education comes from Iran, where researchers conducted a clinical trial comparing SNAPPS to teacher‐centered education.^15^ Thirty‐six midwifery students in their fourth year of midwifery education were divided into groups for their clinical outpatient gynecology rotation. The findings mirrored those from Jain et al^29^ and Shindala et al^30^ in that the group educated in SNAPPS performed better in history taking, developing diagnoses, and creating a management plan of common diseases. The authors credited SNAPPS as a method that views teachers as learning partners and encourages students to develop new knowledge and critical thinking skills.^15^ SNAPPS places emphasis on the student to lead the case presentation. This process also gives the preceptor the opportunity to evaluate the student's knowledge base and to encourage them to step out of their comfort zone by expressing their uncertainties. Because critical thinking is a complex process requiring layers of discovery, SNAPPS pushes the student to lean into uncertainties with the help of their preceptor.^27^ The preceptor can then correct missteps in clinical thinking, while reinforcing positive reasoning and decision‐making.^29^ This in‐the‐moment correcting of errors is considered a hallmark of effective feedback as well as promoting student learning through predictability.^17^, ^22^ Taking a more active role in SNAPPS requires that the student be prepared and engaged in the clinical reasoning process.^20^ More advanced students have consistently been found to benefit more from the challenges of SNAPPS because of their greater knowledge base and experience, but with support and practice, even new learners can benefit from this format.^28^

## DISCUSSION

Although both OMP and SNAPPS are methods that have been used in medical education,^13^ the demand for preceptor training has broadened their application to the advanced practice nursing field.^14^, ^19^ Both methods provide structure for the preceptor process, with improvements in student performance in a broad range of specialties (eg, family, pediatrics, surgery, obstetrics, gynecology).^13^ Although these methods were originally developed for the outpatient setting, their application to th inpatient settings is growing.^13^, ^23^ The OMP method is considered easier to learn and may be more suited to beginning students early in their clinical education, whereas the SNAPPS method is better suited for more advanced learners, challenging them to take control in the clinic.^13^, ^20^, ^28^ Research comparing the models has demonstrated that SNAPPS performed better for clinical reasoning scores, but OMP had a higher user satisfaction rating.^31^ Both methods can be used interchangeably. See Table 1 for a comparison of OMP and SNAPPS.

**Table 1:** Comparison of Clinical Teaching Models

The One‐Minute Preceptor^21^	SNAPPS^27^
Teacher‐led	Learner‐led
1. Get a commitment. The student chooses a diagnosis.	1. Summarize briefly the history and findings.
2. Probe for supporting evidence. The student justifies the diagnosis; preceptor asks for other possibilities.	2. Narrow the differential to 2 to 3 possibilities. The focus is on reasonable diagnostic possibilities.
3. Teach general rules. The preceptor provides pearls.	3. Analyze the differential by comparing and contrasting possibilities. The student considers evidence.
4. Reinforce what was done right. Positive reinforcement is given.	4. Probe the preceptor by asking questions about uncertainties. The student reveals areas of knowledge deficit; the preceptor is knowledge resource.
5. Correct mistakes. Critique the student's performance.	5. Plan the management for the patient's medical issues. The student considers actions for management with preceptor's support.
	6. Select a case‐related issue for self‐directed learning. The student finds a similar case to focus on a specific learning issue.

Abbreviation: SNAPPS, Summarize, Narrow, Analyze, Probe, Plan, Select.

### Application to Midwifery Practice

Both OMP and SNAPPS hold potential for improving and streamlining the work for midwifery preceptors; however, there are obstacles to implementation. Barriers to adapting these methods include the time and financial investment for education programs to incorporate preceptor training into their curriculum. The funding of faculty training, teaching allocation and materials may add to the cost of midwifery education. Midwives themselves may be resistant to these models if the tools are viewed as a medical framework, lacking informed choice and person‐centered care. Additionally, there are insufficient data to reassure midwives that these methods would be a good fit for use in various clinical rotations because the only midwifery study conducted was in a gynecology outpatient setting.^15^ Data are needed to determine the application to hospital inpatient and community birth settings.

Both methods, however, can easily be adopted for midwifery practice by adding a step that specifically addresses informed choice and shared‐decision‐making. For students, education about OMP and SNAPPS can be incorporated into didactic midwifery courses before the student begins clinical rotations. To accommodate the needs of busy midwifery preceptors, online training courses on OMP and SNAPPS methods could be made available, with the addition of continuing education credits as an added incentive. Using laminated cards for each method would help keep preceptors and students on track, improving implementation and time efficiency. Students who had experiences using these methods during their clinical rotations will potentially be better prepared and motivated to become preceptors themselves. Lastly, it is essential that these methods receive rigorous study to validate effectiveness for their use in midwifery practice, as well as in a variety of midwifery practice settings.

## CONCLUSION

As we continue working toward improving obstetric morbidity and mortality in the United States, we must continue educating qualified midwives. To accomplish this, we must also have qualified preceptors using tools that promote critical thinking skills in midwifery students. The use of OMP and SNAPPS as training methods can provide preceptors with a standardized, reliable, and consistent way to engage with midwifery students. In the long run, implementing either method will give preceptors accessible tools that they can use daily to improve student performance and create important positive learning experiences.^31^ Several studies have compared the efficacy of OMP and SNAPPS.^13^, ^20^, ^29^, ^31^ More research is needed, however, to determine the value of these tools for use in midwifery practice. Our profession depends on the growth of a competent midwifery workforce, and these tools are an important step in the path toward success of this goal.

## CONFLICT OF INTEREST

The authors have no conflicts of interest to report.
